# Reactive Oxygen Species and Oxidative Stress in Vascular-Related Diseases

**DOI:** 10.1155/2022/7906091

**Published:** 2022-04-04

**Authors:** Xin-Meng Cheng, Yu-Yuan Hu, Tao Yang, Nan Wu, Xue-Ning Wang

**Affiliations:** Division of Cardiovascular Surgery, Third Hospital of Shanxi Medical University, Shanxi Bethune Hospital, Shanxi Academy of Medical Sciences, Tongji Shanxi Hospital, Taiyuan, Shanxi 030032, China

## Abstract

Oxidative stress (OS) refers to the enhancement of oxidation and the decreased of related antioxidant enzymes activity under pathological conditions, resulting in relatively excess reactive oxygen species (ROS), causing cytotoxicity, which leads to tissue damage and is linked to neurodegenerative diseases, cardiovascular diseases, diabetes, cancers, and many other pathologies. As an important intracellular signaling molecule, ROS can regulate numerous physiological actions, such as vascular reactivity and neuronal function. According to several studies, the uncontrolled production of ROS is related to vascular injury. The growing evidence revealing how traditional risk factors translate into ROS and lead to vasculitis and other vascular diseases. In this review, we sought to mainly discuss the role of ROS and antioxidant mechanisms in vascular-related diseases, especially cardiovascular and common macrovascular diseases.

## 1. Introduction

**Figure 1:**
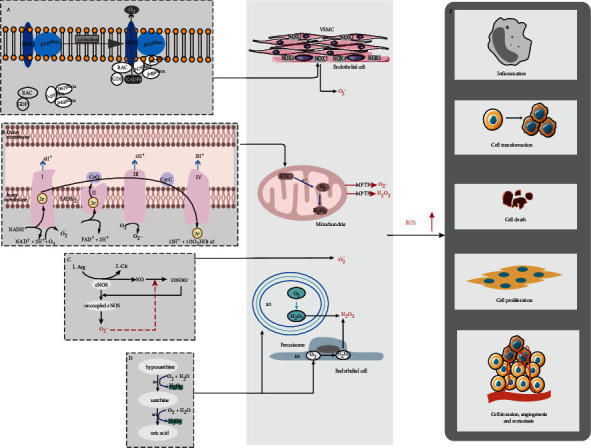
Sources of ROS production. (a) ROS from the NOX2. (b) Electron transfer and ROS generation in ETC. (c) Uncoupled eNOS produced superoxide. (d) OX produced superoxide and hydrogen peroxide by degrading of purines and conversion of hypoxanthine.

Oxidative stress (OS) refers to the imbalance of oxidative metabolism in vivo, mainly due to the enhancement of oxidation, which promotes inflammatory responses and the production of large amounts of oxidative intermediate products, such as free radicals, including superoxide, hydroxyl and nitric oxide, and other ROS, including hydrogen peroxide [1]. ROS formation primarily occurs in the mitochondria and endoplasmic reticulum of eukaryotic cells [2]. Oxidative stress induced by the production of excess ROS has been a pivotal mechanism in cardiovascular diseases [3]. Research shows that ROS can promote the generation of inflammatory cytokines, causing inflammation and impairing the functions of vascular cells by activating transcription factors, upregulating adhesion molecules, stimulating chemokine production, and recruiting inflammatory cells [4, 5]. Based on accumulating evidence, excessive ROS-induced altered vascular functions have been demonstrated, including endothelial cell (ECs) damage, hyperplasia of vascular smooth muscle cells (VSMCs), and structural remodeling [6]. Oxidative stress also plays an important role in vascular diseases by regulating the release of both vasoconstricting and vasodilating factors of ECs and autophagy [7, 8] (Figure 1(e)).

Growing evidences revealing how traditional risk factors translate into ROS and contribute to heart and vascular diseases [9, 10]. Uncontrolled production of ROS is interrelated with vascular injury. We opted to discuss the important intermediate role of oxidative stress in vascular-related diseases, particularly cardiovascular and common macrovascular diseases.

First, we detailed the production of ROS in the vessels and their physiological functions. Second, we investigated the implications of ROS in cardiovascular and common macrovascular diseases. For ECs and VSMCs, oxidative stress can be particularly significant as they can activate multiple pathways to induce either cell proliferation, or migration and death [11, 12]. Considering the importance of oxidative stress in the pathogenesis of vascular dysfunction, in the last part of this review, we explored the potential therapeutic strategies of antioxidant therapy in vascular diseases.

## 2. Sources of Intracellular ROS Production

ROS can play an important role in many intracellular responses. ROS-dependent production in a positive feedback way is the key to continued oxidative stress, which has been linked to interactions between different ROS-producing oxidases systems, and ultimately alters many functions of the vascular cells [13]. The intracellular production of ROS often occurs in the endoplasmic reticulum and mitochondria [14]. As highly reactive molecules, ROS mainly include typical free radicals which contains at least one unpaired electron such as hydroxyl radical (·OH), superoxide (O_2_^−^·), and hydrogen peroxides (H_2_O_2_), and meanwhile, there are adducts such as hypochlorous acid and nitrogen-containing species [15, 16]. O_2_^−^·is the most basic form of ROS produced by cells among them, and others can be produced through O2^−^· metabolism. Under normal circumstances, a handful of ROS are sustainedly produced in cells. However, excessive production of ROS can be caused by the stimulation of pathological factors. Further, failure of the ROS scavenging system is the major cause of oxidative stress. There are multiple sources of ROS, such as NADPH oxidases (NOXs), the mitochondrial electron transport chain (ETC), uncoupled endothelial nitric oxide synthase (eNOS), and xanthine oxidase (XO). ROS overload often occurs due to the intricate relationships between different oxidase components [17–19] (Figure 1).

### 2.1. ROS from the NADPH Oxidase (NOX) Activity

NOX activity is the primary ROS resource [6, 20]. The NOX family consists of seven catalytic subunits, termed NOX1-NOX5 and DUOX1-DUOX2. NOXs are multitransmembrane proteins, and DUOXs are seven-transmembrane proteins which C-terminus exposed to the cytosol. Whereas the function of NOX-derived ROS in vasculature also depends on the cell type. Of these, NOX1 and NOX4 are present in VSMCs, while NOX2 and NOX4 are primarily expressed in ECs [21]. The NOX2 protein is stable when constitutively associated with p22^phox^ [22]. NOX2 can be activated to generate superoxide (O_2_^−^) if the cytosolic components p40^phox^, p47^phox^, and p67^phox^ transferred to the NOX2/p22^phox^ complex, causing the endothelial dysfunction [23–25]. The functional studies of NOX4 have shown that ROS generated by NOX4 is dependent on the p22^phox^ protein, and NOX4 is a high degree of homology to NOX2 [26] (Figure 1(a)).

A portion of NOX-derived ROS are released into the extracellular space which can affect the extracellular matrix (ECM) [27], which another important step in angiogenic response, and participate normal angiogenesis and the development of aortic disease [20, 28].

### 2.2. ROS from the Mitochondrial Electron Transport Chain (ETC)

ROS are produced as by-products of mitochondrial aerobic metabolism, therefore, a mass of ROS are mainly produced via the mitochondrial ETC during normal aerobic metabolism [29]. Based on evidences, approximately 10-fold higher ROS are present in the mitochondrial than in the nucleus and other organelles, and this unmasking explained why mitochondria are primary targets for ROS-induced damage [30].

Complex I is the main intracellular sites of ROS production in the mitochondria and complex III followed. The essential function of complex I is to transfer 2 electrons from reduced NADH to FMN and then to ubiquinone (CoQ) through a series of iron-sulfur centers, where electrons can be generated at both the FMN and CoQ binding sites [31] (Figure 1(b)). As the only reducing electron carrier in complex III, ubisemiquinone can move freely and leak an electron to O_2_, thereby reducing O_2_ to ROS [32, 33].

### 2.3. Xanthine Oxidase (XO)

XO is another major intracellular oxidase. The main functions of XO include the degradation of purines and conversion of hypoxanthine to xanthine which is further converted to uric acid [34]. XOs are found in ECs and plasma and produce superoxide and H_2_O_2_ by donating electrons to molecular oxygen [35–37] (Figure 1(d)).

### 2.4. Uncoupled Endothelial Nitric Oxide Synthase (eNOS)

Studies have shown that eNOS can be activated to produce vascular protective medium, nitric oxide (NO) [5]. NO oxidative inactivation is rapidly induced by excess superoxide, and persistent oxidative stress induces eNOS uncoupling, producing superoxide at the expense of NO. Whereas the decreased NO is a well-established cardiovascular risk factor [35, 38] (Figure 1(c)).

## 3. Oxidative Stress in Common Vascular Diseases

### 3.1. Oxidative Stress in Atherosclerosis

Atherosclerosis (AS) is regarded as a chronic immunoinflammatory disease [39], which is closely related to oxidative stress caused by the production of excess ROS [40]. A generally accepted statement about AS is damage-response theories. Endothelial injury is caused by various stimuli, which causes plasma components such as low-density lipoprotein cholesterol (LDL) to infiltrate and accumulate in the intima, forms atherosclerotic plaques, and leads to narrowing of the vascular lumen [41].

ROS exhibit different mechanisms in the occurrence and development of atherosclerosis. Nonoxidized LDLs by themselves do not risk factors for AS due to its lower affinity for macrophages. When oxidized, LDL becomes oxidized LDL (ox-LDL) in response to ROS [42]. Ox-LDL can stimulate ECs to secrete a variety of inflammatory factors, while promoting vascular cells to secrete ROS by activating NOXs. In particular, ox-LDL increases the expression of vascular endothelial growth factor (VEGF) in macrophages and induces HIF-1*α*, significantly increasing lumen formation in ECs [43, 44]. Consequently, oxidative stress plays a pathogenic role in promoting AS by inducing macrophage infiltration and ox-LDLs to thicken the blood vessel walls [17]. Several decades ago, researchers discovered that ROS are involved in the development of atherosclerotic plaques by oxidizing unsaturated fatty acids in membrane lipids [1, 3]. Foam cells are the result of binding and uptake of ox-LDL by scavenger receptors on the surface of macrophages. In addition, ROS can promote the expression of scavenger receptors in SMCs and their transformation into foam cells [45]. A recent study showed that autophagy deficiency in VSMCs can promote AS by regulating the inflammatory response [46]. These figures suggest that various factors affect the synthesis of ROS directly or indirectly, leading to the occurrence of AS.

Iron is considered as a risk factor for cardiovascular system owing to its ability to catalyze ROS formation [47]. Vinchi and his team were the first to prove the connection between nontransferrin bound serum iron (NTBI) and AS by investigating a mouse model (ApoE-/- FPNwt/C326S), they observed that iron is heavily deposited in the arterial media layer in the presence of elevated NTBI, induces ROS production, apoptosis of SMCs and ECs, and stimulates a large number of MCP-1-mediated monocyte recruitment, formation of unstable plaques, and eventually aggravates atherosclerosis [48, 49]. As a novel pathway of regulated cell death (RCD), the role of ferroptosis in AS is unclear but there is no doubt that ferroptosis is closely related to iron metabolism and promotes oxidative stress leading to cell death [50, 51].

Prolonged estrogen deficiency is an important risk factor for AS in older women [52]. Estrogen plays a direct protective role on the blood vessels by regulating gene expression and function in ECs [53]. Yang et al. successfully induced postmenopausal AS in mouse by feeding the ovariectomized mice a high-fat diet. It has also been confirmed that estrogenic hormones such as dioscin can play an antioxidant role through activating the PGC-1*α*/ER*α* pathway to prevent postmenopausal AS [54]. Notably, chronic infectious disease-associated pathogens have been detected in atherosclerotic plaques, such as pathogenic bacteria including the periodontitis-associated Porphyromonas gingivalis (P. gingivalis). Further, Xie et al. revealed that BMAL1-downregulation and its associated circadian clock disturbance aggravate P. gingivalis-induced atherosclerosis in ApoE-/- mice by elevating oxidative stress formation [55].

### 3.2. Oxidative Stress in Aortic Dissection (AD)

The term “aortic dissection (AD)” refers to the process of intimal tearing within the aortic wall, causing a false lumen and rapid expansion in the aorta. AD is a common clinical type of acute aortic syndrome (AAS), with high mortality and poor prognosis. Hypertension and intimal injury are two important factors in the formation of AD, and atherosclerotic degeneration of aorta is a common precipitating factor for intimal injury, which often leads to intimal tears and the destruction of vascular wall structure [56]. VSMCs and ECs are two main cell types in the vascular system. Therefore, dysfunction of VSMCs and ECs is a key factor in the formation of AD. In pathophysiology, AD is characterized by excessive VSMC loss, ECM degradation, and inflammation. The intima of the aorta consists of collagen, elastin fibers, and a monolayer of ECs. As a result, the aorta is prone to intimal damage by inflammation or physical stimulation, eventually leading to intimal tears [6, 57, 58].

ROS result in ECM remodeling and SMCs apoptosis by activating multiple hypertrophy-signaling kinases and transcription factors and inducing the release of matrix metalloproteinases (MMPs), which leads to ECM remodeling and induces smooth muscle cell apoptosis [59, 60]. Studies have also shown that SMC apoptosis is related to the oxidative stress caused by ribosome biogenesis. Previously, Wu et al. found decreased ribosome biogenesis in aortic-SMCs, which results in apoptosis associated with p53-dependent proliferative inhibition [61]. Based on emerging evidence, autophagy is connected with inflammation, and excessive autophagy leads to the death of ECs, which can result in intimal tear [62]. Consequently, improving endothelial dysfunction, ECM degradation, and SMCs death, all of which can be potential targets for the non-surgical treatment of AD.

**Figure 2:**
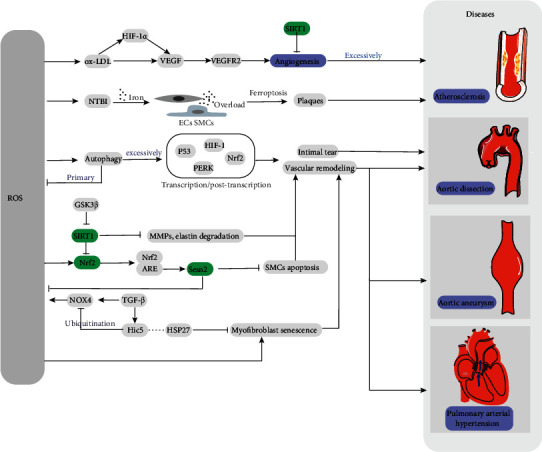
The related mechanism of ROS and diseases.

Liu and Desai previously revealed that transforming growth factor-beta (TGF-*β*) induces myofibroblast differentiation by activating NOX4 to promote ROS production [63]. Meanwhile, TGF-*β*1 induces hydrogen peroxide-inducible clone 5 (Hic-5) expression in a delayed manner, which can bind to heat shock protein HSP27 to counter these effects [64, 65]. Zou et al. showed that HSP27 produced by VSMCs was higher in the aortic wall of AD patients and verified that HSP27 plays a positive role in preventing AD by promoting cell proliferation and inhibiting apoptosis [66]. Accordingly, through further research, Desai et al. discovered that TGF-*β*1-induced Hic-5 degrades NOX4 through a ubiquitination pathway, limiting the senescence of myofibroblasts by a negative feedback mechanism [67]. This is the first study to certify the posttranslational regulation of NOX4 (Figure 2).

Sestrin2 (Sesn2) is an antioxidant protein involved in diseases by regulating levels of oxidative stress [68]. The upregulation of Sesn2 is reported to decrease ox-LDL-induced ROS production and alleviate the apoptosis of macrophage. Sesn2 is also found to be increased in both the aortic tissues and plasma of AD patients. Therefore, through further studies, Xiao et al. found that Sesn2 attenuates Ang II-induced SMCs apoptosis and prevents AD through Nrf2-ARE pathway [69–71].

Potente and his team showed that SIRT1, which belongs to the Sirtuin protein family, is a pivotal protein that regulates angiogenesis. Functional defects of SIRT1 can lead to downregulation of genes involved in vascular development and remodeling, resulting in the vascular-related diseases [72]. Melatonin, one of the hormones secreted by the pineal gland, has been shown to regulate circadian rhythms and play an important role in antioxidative stress and downregulation of MMPs [73]. Xia et al. demonstrated in mouse experiments that melatonin reduces oxidative stress and VSMCs loss by activating SIRT1 signaling in a receptor-dependent manner, which can be a potential therapeutic target in AD [74].

In recent years, researchers have shown that autophagy is important in the pathogenesis of AD. As a common mode of regulated cell death, autophagy is responsible for the degradation of damaged organelles and some proteins which cannot be degraded by proteasomes [75]. Metabolic stress or oxidative stress induces autophagy to degrade potentially harmful ROS-producing organelles, such as the mitochondria. Therefore, autophagy is inherently cytoprotective [76, 77]. Based on emerging evidence, excessive stimulation of autophagy can lead to the death of ECs, which can result in aberrant vascular remodeling or intimal tear. Therefore, whether autophagy is protective or deleterious in AD depends on whether it limits or increases abnormal endothelial proliferation, or whether it prevents normal angiogenesis [50, 78]. Besides of this, autophagy has been confirmed in the lungs of humans with pulmonary arterial hypertension (PAH) and may have a protective effect on endothelial injury that initiates vascular remodeling in PAH [79].

Resveratrol (RES), as a phytoestrogen, is widely found in red wine and grapes and has been proven to have antioxidant properties [80]. Van Andel et al. found that RES can effectively combat oxidative stress by inhibiting NOX production of mitochondrial superoxide, which has a protective effect on the aorta [81].

### 3.3. Oxidative Stress in Aortic Aneurysm (AA)

AA refers to permanent abnormal expansion or localized bulging caused by local pathological weakness of the aortic wall and extension after hypotonia, without intimal tearing. SMCs are the major components of the aortic wall, and their loss through apoptosis or necrosis is a major defining feature of both AA and AD [82]. AA is also characterized by abnormalities in the ECM that compromise the structural integrity of the aorta, and MMPs play a key role in ECM remodeling. Inflammation and ROS are cardinal features of AA. However, ROS in the pathogenesis of AA and AD has received less attention than AS [83–85].

Superoxide dismutase (SOD) is an effective antioxidant enzyme. In fact, zinc can combine with SOD3 as a cofactor to alleviate oxidative stress [86]. Therefore, Zn deficiency plays a crucial role in oxidative damage and inflammatory responses, such as wound healing and homeostasis [87, 88]. Socha et al. determined the mineral content in aorta tissue samples from 108 patients with AA and found that zinc concentration in AA patients was significantly lower than that in healthy subjects [89]. Accordingly, supplementation with antioxidant trace elements in patients with aortic aneurysm may improve the prognosis and mortality of patients.

Nrf2 and its main intracellular regulator, Keap1, function in a pervasive intracellular defense mechanisms to against oxidative stress [90]. Nrf2 can protect cells and restore redox homeostasis by scavenging excessive ROS levels, so it has been proved to have a function of preventing diseases [91, 92]. Kopacz et al. found that inhibiting Nrf2 transcriptional activity in mouse helped AA formation, which can be prevented by simvastatin [93]. Interestingly, inhibition of Nrf2 expression has previously been found to increase the activation of the RAC1-dependent nuclear factor-*κ*B (NF-*κ*B), which means that Nrf2 also plays a critical role in counteracting NF-*κβ*-driven inflammatory responses [94]. Lithium chloride (LiCl) is a compound which can effectively reduce inflammatory response. Xu and his team provided the first evidence that LiCl inhibits ROS production, facilitates the synthesis of elastin, and maintains the stability of aorta structure by regulating the GSK3*β*/SIRT1/NF-*κ*B cascade, ultimately preventing the development of AA [95]. Additionally, LiCl was found to significantly induce SIRT1 expression, which can reduce oxidative stress and VSMC loss to prevent AA [72]. Based on recent evidence, the deficiency of kappa B kinase epsilon (IKK-epsilon) inhibitors also effectively inhibits the generation of ROS, macrophage infiltration, and VSMC apoptosis [96]. Such finding highlights a novel therapeutic strategy for the prevention and treatment of AA by targeting IKK-epsilon.

Aldehyde dehydrogenase 2 (ALDH2) is an enzyme associated with aldehyde metabolism that is mainly located in mitochondria and is closely related to oxidative metabolism of aldehydes produced by lipid peroxidation. Studies have found that lack of ALDH2 can lead to the imbalance between antioxidant defense activity and ROS production, aggravating oxidative stress. Therefore, as an antioxidant gene, ALDH2 activator plays a potential therapeutic role in AA [97, 98].

Heme oxygenase-1 (HO-1) is a cell-protective heme degrading enzyme, which regulates inflammatory responses by limiting intracellular levels of oxyhemoglobin [99]. HO-1 has been reported to attenuate the development of AA in mouse models induced by Ang-II. However, Kopacz et al. revealed the dual role of HO-1 in AA formation, which can prevent the development of AA while simultaneously exacerbating the formation of AA states and reducing the risk of rupture [100, 101].

### 3.4. Oxidative Stress in Pulmonary Arterial Hypertension (PAH)

PAH is a complex degenerative disease and refers to a resting elevated mean pulmonary arterial pressure (≥25 mmHg) and normal wedge pressure (<15 mmHg) [102, 103]. The pathogenesis of PAH is still unclear and is characterized by excessive proliferation of vascular cells and abnormal vascular remodeling. EC dysfunction has been found to produce a variety of endothelial vasoactive mediators, which in turn promote proliferation and migration of VSMCs. And ultimately, hyperproliferation of disordered ECs and VSMCs can induce remodeling of small pulmonary arteries [104–106].

In recent decades, with the development of molecular genetics, the genetics of PAH has evolved rapidly as a research hotspot. Loss of bone morphogenetic protein (BMP) signaling was found to be a major risk factor for PAH development, especially BMPR2 [106]. BMPR2 activates serine/threonine kinases by encoding TGF-*β* II receptors, resulting in transcriptional regulation of phosphorylated Smads. Studies have proved that PAH is caused by mutations in BMPR2 affecting the TGF*β*/Smads signaling pathway [107]. The findings of several recent studies have shown increased DNA damage in lung vascular cells from PAH patients. Federici et al. verified this phenomenon through measurements and found that DNA damage is prevalent in PAHs, which may be genetically related. This study is the first to directly link oxidative stress-induced DNA damage to ROS overload in PAH cells [108].

In the pathogenesis of PAH, the dysfunction of ECs causes homeostasis imbalance of endothelium-derived vasodilator and constrictor factors [105]. Several studies have shown that the generation of endothelium-derived nitric oxide (EDNO) is essential for maintaining vascular homeostasis [5]. Substantial evidence supports that excessive production of ROS directly inactivates EDNO, acts as cell signaling molecules, and promotes protein dysfunction [109]. Phosphodiesterases-5 (PDE-5), which is highly expressed in lung tissue and significant upregulated in PAH, can lead to endothelial dysfunction by inactivating cGMP affecting the vasodilator-NO action. Therefore, the use of PDE inhibitors is of great significance in the treatment of PAH [110, 111].

## 4. Oxidative Stress in Angiogenesis

Although high concentrations of ROS have vascular-damaging effects, oxidative stress has been proved to take an active role in angiogenesis [112]. Typically, ROS derived from NOXs and mitochondrial can induce angiogenesis directly or indirectly [113]. These mechanisms often involve hypoxia-inducible factor/vascular endothelial growth factor (VEGF) signaling, which is essential for normal vascular development and has great potential in wound repair and the treatment of ischemic diseases [114, 115].

## 5. New Advances in Antioxidant Therapy

In summary, excessive generation of ROS or failure of the ROS clearance system is the main factor causing ROS accumulation. Consequently, inhibiting the generation of ROS and promoting the metabolism of ROS are essential for the antioxidative stress response. Xie et al. revealed that CoQ10 can affect mitochondrial function, inhibit ETC-derived ROS production, and control oxidative stress responses by activating the AMPK/YAP/OPA1 pathway [116]. It has been established that the antioxidant system plays a key role in the process of antioxidative stress (Table 1).

The antioxidant system can protect tissues from ROS toxicity, which includes antioxidant enzymes such as superoxide dismutase (SODs), catalase and glutathione peroxidase (GSH-Px), and nonenzymatic antioxidants such as bilirubin, *α*-tocopherol, and *β*-carotene [117].

Among them, SODs are the most potent antioxidant enzymes and are metalloproteins which catalyze the conversion of superoxide anions (•O^2−^) into H_2_O_2_. Three isoforms of SOD required different cofactors to assist their activities: copper (Cu) and zinc (Zn) are cofactors for SOD1 and SOD3, whereas the SOD2 tetramer requires manganese (Mn) as a cofactor [118]. The average concentrations of Zn and Cu in the aorta of patients with AA were significantly lower than those in the aorta samples of healthy individuals [89]. Therefore, supplementation of micronutrients with antioxidant properties such as Zn and Cu in the perioperative period can improve the prognosis of surgery to a certain extent.

Catalase and Gpx convert hydrogen peroxide into water, and Gpx has reducing properties. Vitamin C, which is known as ascorbic acid, is also recognized to be the most effective antioxidant in plasma [119]. As a lipophilic vitamin, vitamin E exerts a powerful antioxidant effect by preventing lipid peroxidation.

## 6. Conclusion and Perspectives

In this review, we introduce the mechanism of ROS production and analyze the role of oxidative stress responses in the pathogenesis of AS, AD, AA, and PAH in detail. As mentioned previously, the imbalance between prooxidant and antioxidant systems results in the excessive production of ROS, which affects the inflammatory response and cell proliferation, death and transformation through various pathways, leading to the occurrence of a variety of common diseases (Figure 1). Aortic diseases are life-threatening conditions with no effective medicines for the treatment, which mainly include aortic open surgery or endovascular replacement.

At present, studies have confirmed that ROS can regulate the proliferation, apoptosis, extracellular matrix remodeling, and intima damage of vascular cells through a variety of classical molecules and pathways, such as Nrf2, TGF-*β*, and the novel pathways of regulated cell death such as autophagy and ferroptosis also play an important role, thus causing the occurrence of a variety of vascular diseases (Figure 2). As biomarkers of these diseases, well-controlled ROS may be beneficial for preventing cardiovascular disease and promoting angiogenesis during tissue repair. Therefore, our research is mainly devoted to the prevention, improvement of the prognosis, and effective nonsurgical therapies for such diseases via the identification of targets for antioxidant drugs.

As we all know, as the most common dietary antioxidant compounds, vitamin A, C, E, and other trace elements, phenolic compounds, and carotenoids are abundantly found in plants and served as a safe way to prevent diseases. In addition, as effective antioxidants, antioxidant enzymes and their cofactors, such as zinc and copper, have also been extensively studied and play a great potential in the prevention and treatment of diseases.

## Figures and Tables

**Table 1:** Role of antioxidants on vascular diseases.

Antioxidants	Effect on antioxidant system	Reference
CoQ10	Activating AMPK-YAP-OPA1 pathway, decrease mitochondrial superoxide	116
Resveratrol (RES)	As a phytoestrogen, decrease NOX, mitochondrial superoxide	54,81
Zn	Combines with SOD3 as a cofactor	86-88
Catalase	Detoxify hydrogen peroxide into water	117
GSH-Px	Reducing properties	117
Statins	Decrease NOX, prevent eNOs uncoupling	93
Lithium chloride (LiCl)	Regulation GSK3*β*/SIRT1/NF-*κ*B cascade, decrease inflammation, MMPs, and superoxide	72,95
*β*-Carotene	*β*-Carotene	117
Vitamin C	Water-soluble antioxidant	119
Vitamin E	Activated to *α*-tocopherol, preventing lipid per-oxidation	117
Melatonin	Via activation of SIRT1 signaling in a receptor-dependent manner.	69
